# Antibiotic susceptibility patterns and trends of the gram-negative bacteria isolated from the patients in the emergency departments in China: results of SMART 2016–2019

**DOI:** 10.1186/s12879-024-09294-0

**Published:** 2024-05-17

**Authors:** Ying Fu, Feng Zhao, Jie Lin, Pengcheng Li, Yunsong Yu

**Affiliations:** 1 Department of Clinical Laboratory, Sir Run Run Shaw Hospital, School of Medicine, Zhejiang University, 310016 Hangzhou, Zhejiang Province China; 2 Key Laboratory of Precision Medicine in Diagnosis and Monitoring Research of Zhejiang Province, 310016 Hangzhou, Zhejiang Province China; 3 MRL Global Medical Affairs, MSD China, 200233 Shanghai, China; 4 Department of Infectious Diseases, Sir Run Run Shaw Hospital, School of Medicine, Zhejiang University, 310016 Hangzhou, Zhejiang Province China; 5 Key Laboratory of Microbial Technology and Bioinformatics of Zhejiang Province, 310012 Hangzhou, Zhejiang Province China; 6 Regional Medical Center for National Institute of Respiratory Diseases, Sir Run Run Shaw Hospital, School of Medicine, Zhejiang University, 310016 Hangzhou, Zhejiang Province China

**Keywords:** SMART, Carbapenem, OSWIA, Antibiogram, Cephalosporin, Antibiotic-Resistance

## Abstract

**Background:**

The study aims were to evaluate the species distribution and antimicrobial resistance profile of Gram-negative pathogens isolated from specimens of intra-abdominal infections (IAI), urinary tract infections (UTI), respiratory tract infections (RTI), and blood stream infections (BSI) in emergency departments (EDs) in China.

**Methods:**

From 2016 to 2019, 656 isolates were collected from 18 hospitals across China. Minimum inhibitory concentrations were determined by CLSI broth microdilution and interpreted according to CLSI M100 (2021) guidelines. In addition, organ-specific weighted incidence antibiograms (OSWIAs) were constructed.

**Results:**

*Escherichia coli* (*E. coli*) and *Klebsiella pneumoniae* (*K. pneumoniae*) were the most common pathogens isolated from BSI, IAI and UTI, accounting for 80% of the Gram-negative clinical isolates, while *Pseudomonas aeruginosa* (*P. aeruginosa*) was mainly isolated from RTI. *E. coli* showed < 10% resistance rates to amikacin, colistin, ertapenem, imipenem, meropenem and piperacillin/tazobactam. *K. pneumoniae* exhibited low resistance rates only to colistin (6.4%) and amikacin (17.5%) with resistance rates of 25–29% to carbapenems. *P. aeruginosa* exhibited low resistance rates only to amikacin (13.4%), colistin (11.6%), and tobramycin (10.8%) with over 30% resistance to all traditional antipseudomonal antimicrobials including ceftazidime, cefepime, carbapenems and levofloxacin. OSWIAs were different at different infection sites. Among them, the susceptibility of RTI to conventional antibiotics was lower than for IAI, UTI or BSI.

**Conclusions:**

Gram-negative bacteria collected from Chinese EDs exhibited high resistance to commonly used antibiotics. Susceptibilities were organ specific for different infection sites, knowledge which will be useful for guiding empirical therapies in the clinic.

**Supplementary Information:**

The online version contains supplementary material available at 10.1186/s12879-024-09294-0.

## Background

Antimicrobials are frequently used in emergency departments (EDs) in China and a study noted that the proportion of emergency patients treated with antibiotics was as high as 39.31 to 43.45% from 2016 to 2019 [1]. Antibiotic stewardship in EDs should avoid administration of broad-spectrum antibiotics, shorten their use times, as well as minimizing their unnecessary use [2]. However, as patients presenting to EDs are often in an acute state, physicians have to make decisions in a very short time frame and they prescribe antibiotics empirically. For critically ill infected patients, guidelines recommend to start antibiotic treatment in the first hour of recognition [3], which means that it is frequently impossible to get microbiology results to guide the choice of antimicrobial therapy. In order to support the choice of empiric antibiotic treatments, consensus guidelines as well as local antibiotic drug susceptibility detection and various antimicrobial surveillance programs have been introduced in China [4, 5].

One approach to individualized empiric antibiotic therapy is the Weighted-Incidence Syndromic Combination Antibiogram, which is comprised of information about the likelihood a treatment regimen will be effective for all relevant organisms for a given infection based on existing large datasets [6–8]. A similar approach is the organ-specific weighted incidence antibiogram (OSWIA), which estimates probable susceptibilities of organ specific isolates to specific antibiotics [9]. The Study for Monitoring Antimicrobial Resistance Trends (SMART) global surveillance program monitors *in vitro* susceptibilities of clinical Gram-negative bacilli to antimicrobial agents obtained from blood stream infections (BSI), intra-abdominal infections (IAI), urinary tract infections (UTI) and respiratory tract infections (RTI). The purpose of the present study was to determine the prevalence and susceptibilities of various bacteria to conventional antibiotics in patients attending Chinese EDs through a retrospective analysis of the SMART data collected from 2016 to 2019 and to determine differences of organ distributions between the infecting bacterial strains.

## Methods

### Ethics

In this study, the patient informed consent was waived and authorized by the Ethics Committee of Sir Run Run Shaw Hospital, Zhejiang University School of Medicine (Approval Number: 20210811-33).

### Isolates

All bacterial isolates were collected from discarded clinical specimens of hospitalized patients with BSI, IAI, UTI and RTI between 2016 and 2019 who were admitted to the EDs of 18 hospitals across China (Supplementary Table 1). The IAI specimen is derived from tissues or organs within the abdominal cavity, including the stomach, intestines, liver, spleen, pancreas, kidneys that have been infected by pathogens, resulting in infectious diseases [10]. RTI refers to an infection of the tissues in the respiratory system by pathogens such as viruses, bacteria, or fungi. RTI specimens from the respiratory tract included nasal and throat swabs, sputum samples, bronchoalveolar lavage fluid, respiratory secretions, and others [11]. Identification of isolates was initially made by each hospital laboratory and then the specimens were sent for laboratory re-identification using MALDI-TOF/MS (Bruker Daltonics, USA). Any duplicate isolates collected from the same patient were excluded from the data analysis.

### Antimicrobial susceptibility testing

Testing was carried out in the Peking Union Medical College Hospital clinical microbiology laboratory using the Trek Diagnostic System (Thermo Fisher Scientific). Clinical isolates and reference strains were detected using the microbroth dilution method. Minimum inhibitory concentrations (MICs) were determined with reference to the antimicrobial breakpoint of the CLSI M100 (2021) [12]. The antibiotics tested were amikacin (AMK), cefepime (FEP), ceftazidime (CAZ), aztreonam (ATM), ceftriaxone (CRO), colistin (COL), ertapenem (ETP), levofloxacin (LVX), cefoxitin (FOX), imipenem (IPM), tobramycin (TOB), meropenem (MEM) and piperacillin–tazobactam (TZP).

### Definition of antimicrobial-resistant strains

Carbapenem resistance of *Escherichia coli* (*E. coli*) and *Klebsiella pneumoniae* (*K. pneumoniae*) refers to resistance to any of IPM, MEM or ETP. Carbapenem-resistant *Pseudomonas aeruginosa* (*P. aeruginosa*) was defined as resistance to IPM or MEM.

Quinolone resistance to *E. coli, K. pneumoniae* and *P. aeruginosa* was defined as resistance to LVX.

Third generation cephalosporin resistance to *E. coli* and *K. pneumoniae* was defined as resistance to any CRO or CAZ, and the strains susceptible or intermediate to carbapenems (IPM, MEM or ETP). *P. aeruginosa* resistant to third-generation cephalosporin was defined as resistance to CAZ (CAZ-resistant PA) and the strains susceptible or intermediate to carbapenems (IPM, MEM).

### OSWIA calculation

Data were retrospectively analyzed to establish the distribution of bacteria in various organs for BSI, IAI, UTI and RTI. OSWIA values were determined as previously described [9].

## Results

### Patient characteristics and specimen source

 Between January 01, 2016 and December 31, 2019, a total of 656 isolated were obtained from ED patients. The patient characteristics are detailed in Table 1. The patients average age was 60.6 years (range: 1–101), comprising 388 males and 268 females. Most infections were hospital-acquired (HA) (58.1%), while 249 (38.0%) were community-acquired (CA) and for 26 data were not applicable. The isolates included 210 strains from IAI collected during surgery from the peritoneal fluid, appendix, abscesses, pancreas, gall bladder, liver and stomach. A total of 122 strains from BSI, 112 strains from UTI mainly from the urine, and 208 strains from RTI taken from bronchoalveolar lavage, endotracheal aspirate, thoracentesis or sputum were identified, as well as 4 strains from unconfirmed organs.

**Table 1:** Patient characteristics

Variable	Patient (*N* = 656), *n* (%)
Gender	
Male	388 (59.1)
Female	268 (40.9)
Average age (range), years	60.6 (1–101)
Age categories, years	
≤ 39	104 (15.9)
40-59	173 (26.4)
≥ 60	379 (57.8)
Timing of infection onset	
Community-acquired	249 (38.0)
Hospital-acquired	381 (58.1)
Not applicable	26 (4.0)
Specimen source	
BSI: Blood	122 (18.6)
IAI	210 (32.0)
Abscess	26 (4.0)
Appendix	100 (15.2)
Gall bladder	29 (4.4)
Liver	15 (2.3)
Pancreas	2 (0.3)
Peritoneal fluid	36 (5.5)
Stomach	1 (0.2)
Other	1 (0.2)
UTI	112 (17.1)
Ureter	3 (0.5)
Urine	109 (16.6)
RTI	208 (31.7)
Bronchoalveolar lavage	8 (1.2)
Endotracheal aspirate	2 (0.3)
Sputum	196 (29.9)
Thoracentesis	2 (0.3)
Unconfirmed infection	4 (0.6)

*Abbreviations:* *BSI* blood steam infection, *IAI* intra-abdominal infection, *RTI* respiratory tract infection, *UTI* urinary tract infection

### Distribution of Gram-negative bacteria obtained from BSI, IAI, UTI and RTI

**Fig. 1:**
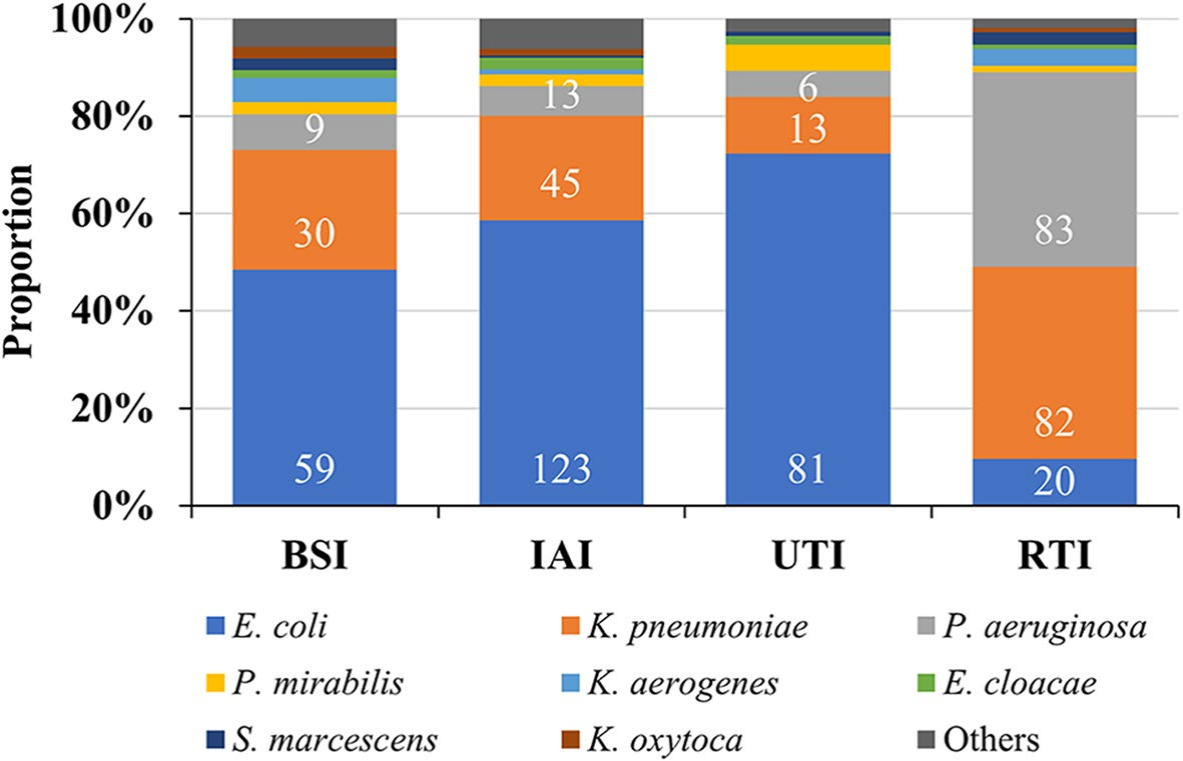
Distribution of Gram-negative bacilli in BSI, IAI, UTI and RTI. Abbreviations: BSI, blood steam infection; IAI, intra-abdominal infection; UTI, urinary tract infection

Enterobacterales were the most common Gram-negative bacilli isolated from emergency patients with BSI, IAI and UTI (Fig. 1). *E. coli* accounted for 48.4% in BSI, 58.6% in IAI and 72.3% in UTI, while *K. pneumoniae* accounted for 24.6%, 21.4% and 11.6%, respectively and other *Enterobacterales* were much less common than *E. coli* and *K. pneumoniae*. The pathogen distribution in RTI was distinctly different from the other three infection types, with *P. aeruginosa* and *K. pneumoniae* being the most common species each accounting for about 40% of the Gram-negative pathogens. Since the composition ratio of Gram-negative bacteria was different at different infection sites (Fig. 1, Supplementary Table 2) the varying patterns between infected organs should be considered when prescribing empirical treatments.

### Distribution of Gram-negative bacteria from 2016 to 2019

**Fig. 2:**
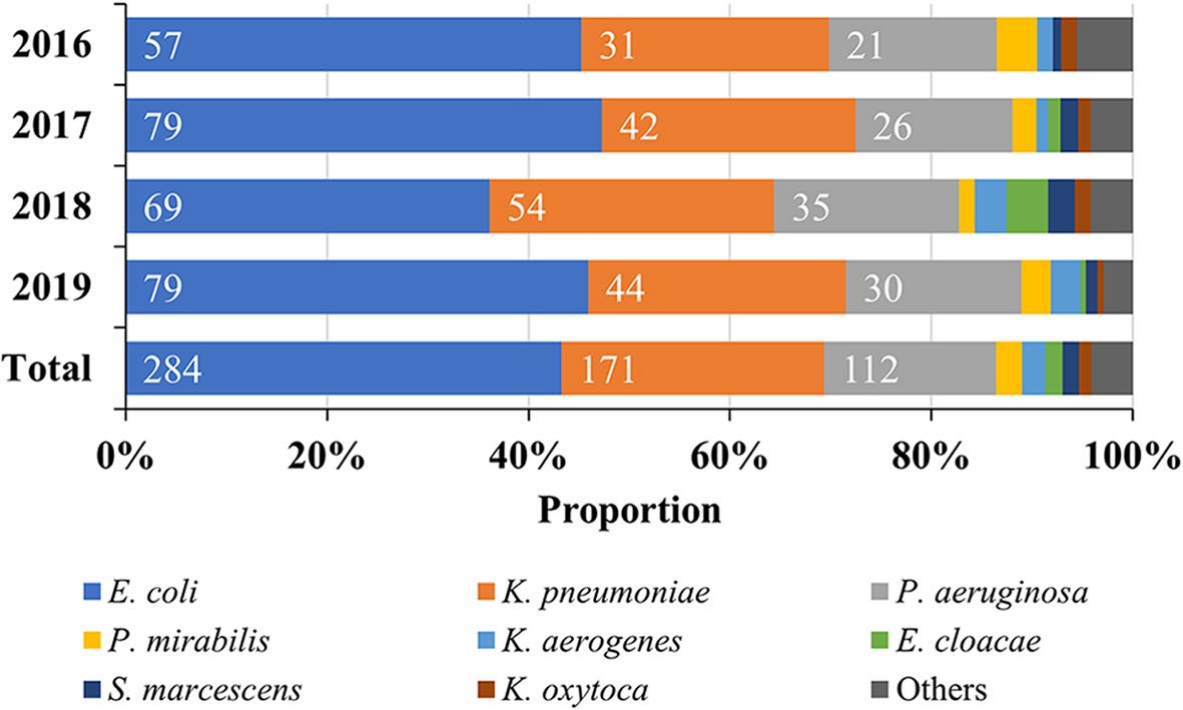
Composition ratio of Gram-negative bacteria in EDs from 2016 to 2019.  Abbreviation: ED, emergency department.

The distribution of Gram-negative pathogens was stable between 2016 and 2019, with *E. coli*, *K. pneumoniae* and *P. aeruginosa* being the top 3 species, accounting for more than 80% of the clinical isolates (Fig. 2, Supplementary Table 3).

### Distribution of Gram-negative bacteria in different age groups of patients

**Fig. 3:**
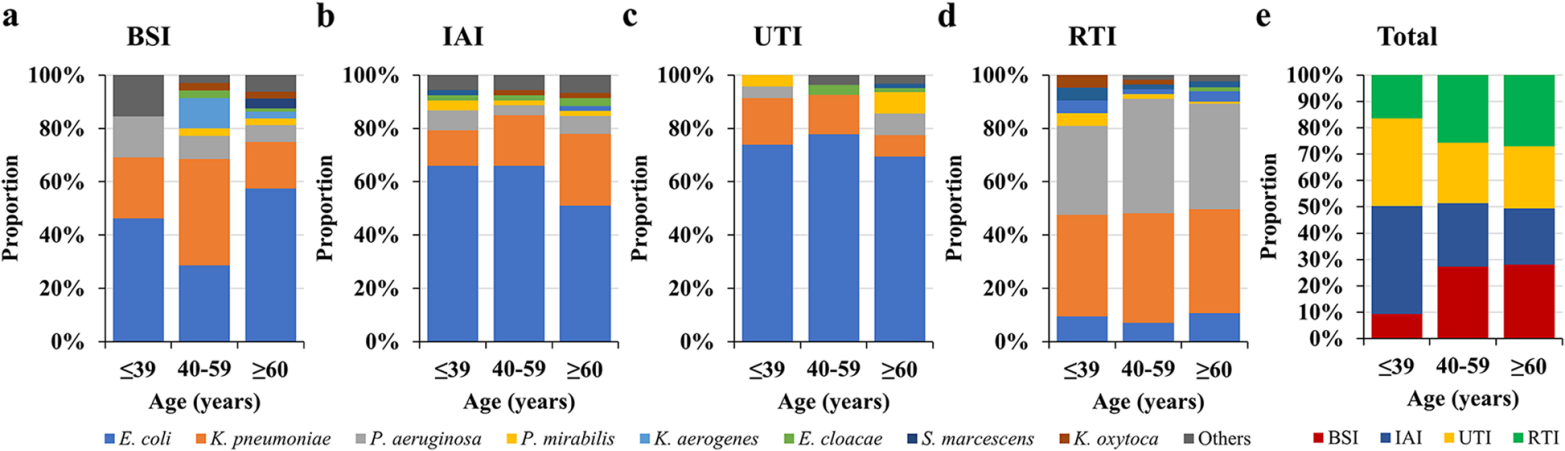
Comparison of the composition ratio of microbiota in different infected organs and age groups.  Abbreviations: BSI, blood steam infection; IAI, intra-abdominal infection; RTI, respiratory tract infection; UTI, urinary tract infection.

Among the strains collected, regardless of the organ of origin, the predominant species were *P. aeruginosa, E. coli* and *K. pneumoniae*. However, the composition ratio of the main bacterial groups were different in infection sites within age groups (Fig. 3a-d) and were generally different especially in the age group ≤ 39 years (Fig. 3e, Supplementary Table 4).

### Monitoring of drug susceptibility

#### Drug resistance rate monitoring of major Gram-negative bacteria from 2016 to 2019

*E. coli* exhibited < 10% resistance to AMK, COL, ETP, IPM, MEM and TZP, with the exception in 2016 to TZP, but generally TZP resistance rates were reduced between 2016 and 2019. Otherwise, resistance rates were more than 30%, with the exception of FOX (16.2%). *K. pneumoniae* exhibited < 20% resistance only to AMK and 6.4% to COL between 2016 and 2019. *P. aeruginosa* only exhibited low resistant rates of 13.4% to AMK, 11.6% to COL and 10.8% to TOB from 2016 to 2019 (Table 2).

**Table 2:** * In vitro* antimicrobial resistance rates of major Gram-negative bacteria (*E. coli*, *K. pneumoniae*, *P. aeruginosa*) from 2016 to 2019 (%)

	AMK	ATM	CAZ	COL	CRO	ETP	FEP	FOX	IPM	LVX	MEM	TOB	TZP
***E. coli* (*n* = 284)**	**7 (2.5%)**	**51 (45.5%)**	**88 (31.0%)**	**16 (5.6%)**	**162 (57.0%)**	**12 (4.2%)**	**127 (44.7%)**	**46 (16.2%)**	**8 (2.8%)**	**161 (56.7%)**	**9 (3.2%)**	**-**	**26 (9.2%)**
2016 (*n* = 57）	3 (5.3%)	10 (47.6%)	27 (47.4%)	5 (8.8%)	41 (71.9%)	5 (8.8%)	37 (64.9%)	15 (26.3%)	4 (7.0%)	35 (61.4%)	5 (8.8%)	-	10 (17.5%)
2017 (*n* = 79)	2 (2.5%)	13 (50.0%)	16 (20.3%)	5 (6.3%)	39 (49.4%)	4 (5.1%)	33 (41.8%)	16 (20.3%)	3 (3.8%)	43 (54.4%)	3 (3.8%)	-	7 (8.9%)
2018 (*n* = 69)	1 (1.4%)	17 (48.6%)	20 (29.0%)	3 (4.3%)	38 (55.1%)	2 (2.9%)	26 (37.7%)	7 (10.1%)	1 (1.4%)	39 (56.5%)	1 (1.4%)	-	5 (7.2%)
2019 (*n* = 79)	1 (1.3%)	11 (36.7%)	25 (31.6%)	3 (3.8%)	44 (55.7%)	1 (1.3%)	31 (39.2%)	8 (10.1%)	0 (0.0%)	44 (55.7%)	0 (0.0%)	-	4 (5.1%)
***K. pneumoniae *(*n*** **= 171)**	**30 (17.5%)**	**125 (44.0%)**	**63 (36.8%)**	**11 (6.4%)**	**76 (44.4%)**	**49 (28.7%)**	**63 (36.8%)**	**66 (38.6%)**	**44 (25.7%)**	**69 (40.4%)**	**43 (25.1%)**	**-**	**50 (29.2%)**
2016 (*n* = 31)	7 (22.6%)	35 (61.4%)	11 (35.5%)	3 (9.7%)	14 (45.2%)	11 (35.5%)	11 (35.5%)	13 (41.9%)	8 (25.8%)	14 (45.2%)	8 (25.8%)	-	8 (25.8%)
2017 (*n* = 42)	8 (19.0%)	26 (32.9%)	19 (45.2%)	2 (4.8%)	23 (54.8%)	13 (31.0%)	22 (52.4%)	20 (47.6%)	13 (31.0%)	20 (47.6%)	13 (31.0%)	-	15 (35.7%)
2018 (*n* = 54)	7 (13.0%)	29 (42.0%)	16 (29.6%)	3 (5.6%)	23 (42.6%)	10 (18.5%)	16 (29.6%)	14 (25.9%)	10 (18.5%)	19 (35.2%)	9 (16.7%)	-	10 (18.5%)
2019 (*n* = 44)	8 (18.2%)	35 (44.3%)	17 (38.6%)	3 (6.8%)	16 (36.4%)	15 (34.1%)	14 (31.8%)	19 (43.2%)	13 (29.5%)	16 (36.4%)	13 (29.5%)	-	17 (38.6%)
***P. aeruginosa *(*n* = 112)**	**15 (13.4%)**	**51 (45.5%)**	**39 (34.8%)**	**13 (11.6%)**	**N**	**N**	**37 (33.0%)**	**N**	**44 (39.3%)**	**41 (36.6%)**	**40 (35.7%)**	**7 (10.8%)**	**42 (37.5%)**
2016 (*n* = 21)	4 (19.0%)	10 (47.6%)	9 (42.9%)	2 (9.5%)	N	N	7 (33.3%)	N	11 (52.4%)	6 (28.6%)	9 (42.9%)	-	10 (47.6%)
2017 (*n* = 26)	5 (19.2%)	13 (50.0%)	11 (42.3%)	1 (3.8%)	N	N	11 (42.3%)	N	9 (34.6%)	12 (46.2%)	9 (34.6%)	-	9 (34.6%)
2018 (*n* = 35)	3 (8.6%)	17 (48.6%)	10 (28.6%)	5 (14.3%)	N	N	12 (34.3%)	N	15 (42.9%)	15 (42.9%)	15 (42.9%)	5 (14.3%)	13 (37.1%)
2019 (*n* = 30)	3 (10.0%)	11 (36.7%)	9 (30.0%)	5 (16.7%)	N	N	7 (23.3%)	N	9 (30.0%)	8 (26.7%)	7 (23.3%)	2 (6.7%)	10 (33.3%)

*N* natural drug resistance, -, no detection

*Abbreviations:* *AMK* amikacin, *ATM* aztreonam, *CAZ* ceftazidime, *COL* colistin, *CRO* ceftriaxone, *ETP* ertapenem,*FEP* cefepime, *FOX* cefoxitin, *IPM* imipenem, *LVX* levofloxacin, *MEM* meropenem, *TOB* tobramycin, *TZP* piperacillin-tazobactam

### Detection rate and drug susceptibility of specific antibiotic-resistant bacteria from 2016 to 2019

#### Isolation of carbapenem-resistant, quinolone-resistant or third-generation cephalosporin-resistant *E. coli, K. pneumoniae and P. aeruginosa*

**Fig. 4:**
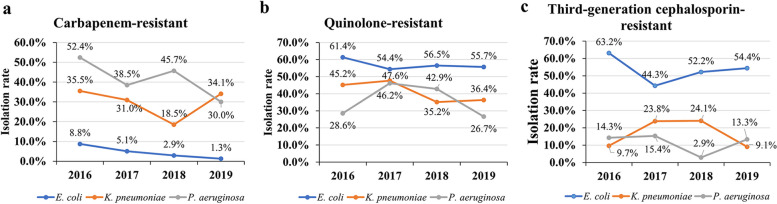
Isolation (detection) rate of carbapenem-resistant, quinolone-resistant, third-generation cephalosporin-resistant *E. coli,* *K. pneumoniae *and* P. aeruginosa* from 2016 to 2019

In isolates, the carbapenem-resistant *E. coli and P. aeruginosa *showed an overall downward trend. However, the rate of detection of carbapenem-resistant *E. coli *was relatively low, being only 1.3% in 2019, while for carbapenem-resistant *P. aeruginosa *in 2019 it was 30.0% and for carbapenem-resistant *K. pneumoniae* strains in 2019, 34.1%, the rate being higher than in 2018 (18.5%). The rate of detection of quinolone-resistant *E. coli *or *K. pneumoniae* showed a decreasing trend in the four years studied, being 55.7% in 2019 for the detection of quinolone-resistant *E. coli *and 36.4% for *K. pneumoniae*. The detection rate of quinolone-resistant *P. aeruginosa* was 26.7% in 2019 and lower than in 2018 (42.9%). The detection rates of third-generation cephalosporin-resistant *E. coli *and *K. pneumoniae* as well as *P. aeruginosa* showed an irregular trend from 2016 to 2019, being between 44.3%–63.2% and 9.1%–24.1% as well as 2.9%–15.4%, respectively throughout the years. Compared to *E. coli, *there were only few numbers of third-generation cephalosporin-resistant *K. pneumoniae* and *P. aeruginosa* isolates found between 2016 and 2019 (Fig. 4, Supplementary Table 5).

#### Specific drug resistance rates (%) of strains to antibiotics from 2016 to 2019

For *E. coli*, the resistance rate of carbapenem-resistant *E. coli *to AMK was 33.3%, and to the other antibiotics tested were > 60%, apart from COL (25.0%). Quinolone-resistant *E. coli *exhibited the lowest resistance rates to AMK (4.3%), ETP (5.6%), IPM (5.0%) and MEM (5.6%). The resistance rates of third-generation cephalosporin-resistant *E. coli *was 2.0% to AMK and 0% to ETP, IPM and MEM.

Carbapenem-resistant *K. pneumoniae* were 57.1% resistant to AMK and quinolone-resistant *K. pneumoniae* were 42.0% resistant to AMK, with low resistance rates only found to COL (8.2% and 10.1%), respectively. Third-generation cephalosporin-resistant *K. pneumoniae* was 6.7% resistant to AMK and 0% to ETP, IPM and MEM.

For carbapenem-resistant *P. aeruginosa*, the drug resistance to AMK was 30.4%, for TOB 28.0% and for COL (10.9%). For quinolone-resistant *P. aeruginosa, *only resistance rates to AMK (24.4%) and TOB (26.1%) as well as COL (9.8%) remained low. Third-generation cephalosporins resistant *P. aeruginosa,*showed low resistance rates of 0% to AMK, IPM, MEM and TOB (Table 3).

**Table 3:** Drug-resistant rates (%) of carbapenem*-*resistant, quinolone-resistant*, *third-generation cephalosporin*-*resistant *E. coli, K. pneumoniae *and *P. aeruginosa *between 2016 and 2019 (n/%)

	*E. coli*			*K. pneumoniae*			*P. aeruginosa*		
	Carbapenem - resistant (*n* = 12)	Quinolone - resistant (*n* = 161)	Third generation cephalosporin - resistant (*n* = 150)	Carbapenem - resistant (*n* = 49)	Quinolone - resistant (*n* = 69)	Third generation cephalosporin - resistant (*n* = 30)	Carbapenem - resistant (*n* = 46)	Quinolone - resistant (*n* = 41)	Third generation cephalosporin - resistant (*n* = 12)
AMK	4 (33.3%)	7 (4.3%)	3 (2.0%)	28 (57.1%)	29 (42.0%)	2 (6.7%)	14 (30.4%)	10 (24.4%)	0 (0.0%)
ATM	11 (91.7%)	98 (60.9%)	113 (75.3%)	47 (95.9%)	61 (88.4%)	19 (63.3%)	31 (67.4%)	33 (80.5%)	11 (91.7%)
CAZ	9 (75.0%)	69 (42.9%)	79 (52.7%)	45 (91.8%)	59 (85.5%)	18 (60.0%)	27 (58.7%)	25 (61.0%)	12 (100.0%)
COL	3 (25.0%)	10 (6.2%)	7 (4.7%)	4 (8.2%)	7 (10.1%)	4 (13.3%)	5 (10.9%)	4 (9.8%)	1 (8.3%)
CRO	12 (100.0%)	117 (72.7%)	150 (100.0%)	49 (100.0%)	65 (94.2%)	27 (90.0%)	N	N	N
ETP	12 (100.0%)	9 (5.6%)	0 (0.0%)	49 (100.0%)	45 (65.2%)	0 (0.0%)	N	N	N
FEP	9 (75.0%)	94 (58.4%)	118 (78.7%)	45 (91.8%)	58 (84.1%)	18 (60.0%)	30 (65.2%)	25 (61.0%)	7 (58.3%)
FOX	11 (91.7%)	31 (19.3%)	21 (14.0%)	49 (100.0%)	58 (84.1%)	14 (46.7%)	N	N	N
IPM	8 (66.7%)	8 (5.0%)	0 (0.0%)	44 (89.8%)	43 (62.3%)	0 (0.0%)	44 (95.7%)	25 (61.0%)	0 (0.0%)
LVX	9 (75.0%)	161 (100.0%)	108 (72.0%)	45 (91.8%)	69 (100.0%)	21 (70.0%)	27 (58.7%)	41 (100.0%)	6 (50.0%)
MEM	9 (75.0%)	9 (5.6%)	0 (0.0%)	43 (87.8%)	43 (62.3%)	0 (0.0%)	40 (87.0%)	27 (65.9%)	0 (0.0%)
TOB	-	-	-	-	-	-	7 (28.0%)	6 (26.1%)	0 (0.0%)
TZP	9 (75.0%)	22 (13.7%)	15 (10.0%)	45 (91.8%)	48 (69.6%)	4 (13.3%)	32 (69.6%)	26 (63.4%)	9 (75.0%)

*N* natural drug resistance; -, no detection

*Abbreviations:* *AMK* amikacin, *ATM* aztreonam, *CAZ* ceftazidime, *COL* colistin, *CRO* ceftriaxone, *ETP* ertapenem,*FEP* cefepime, *FOX* cefoxitin, *IPM* imipenem, *LVX* levofloxacin, *MEM* meropenem, *TOB* tobramycin, *TZP* piperacillin-tazobactam

### Antimicrobial susceptibility monitoring during empiric treatment of different infection sites and organs

**Fig. 5:**
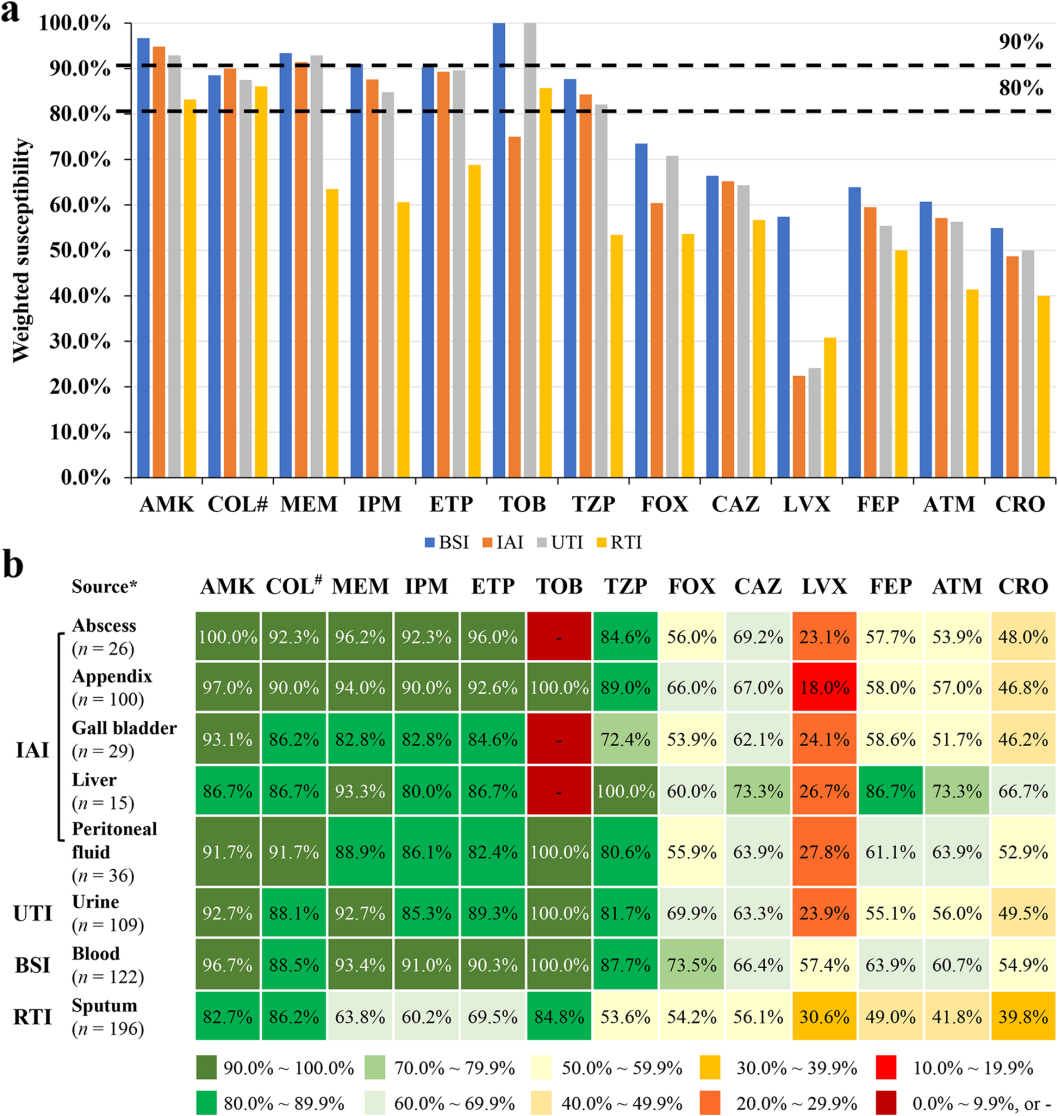
Organ distribution related susceptibilities. **a **Differences in susceptibility of antibiotics at different infection sites. **b** Differences in weighted drug susceptibilities of antibiotics at different organs and infection sites. Note: *only sites from which more than 10 pathogenic bacteria were collected have been included; #, intermediate rate was shown for COL; -, no detection. Abbreviations: AMK, amikacin; ATM, aztreonam; BSI, blood steam infections; CAZ, ceftazidime; CRO, ceftriaxone; ETP, ertapenem; FEP, cefepime; FOX, cefoxitin; IAI, intra-abdominal infections; IPM, imipenem; LVX, levofloxacin; MEM, meropenem; RTI, respiratory tract infections; TOB, tobramycin; TZP, piperacillin/tazobactam; UTI, urinary tract infections

In the weighted susceptibility assessment of different infection sites, it was found that the susceptibility of the same antibacterial drug at different organs and infection sites was different. For example, AMK, TOB, ETP, IPM and MEM were the antibiotics with > 90% susceptibility for BSI, but only AMK and MEM were > 90% effective antibiotics against IAI. High-susceptibility to antibiotics in UTI included AMK, TOB and MEM (all > 90%), and except for AMK, COL and TOB, the susceptibility to other antibiotics at the site of RTI infections was < 80% (Fig. 5, Supplementary Table 6).

## Discussion

This study analyzed Chinese data from the global SMART surveilance program and found that the most frequently isolated Gram-negative bacteria were *Enterobacterales*, a finding similar to previous results from SMART studies and the China Antimicrobial Surveillance Network (CHINET) [13, 14]. *Enterobacterales* are of particular concern given their ability to develop and spread resistance to penicillins, cephalosporins, carbapenems and quinolones [12, 15, 16]. As these are the most commonly used antibiotics in hospitals, such resistance would leave physicians with very limited treatment options. The vast majority of the pathogens were *E. coli*, *K. pneumoniae* and *P. aeruginosa *(86.4%), with resistance rates for cephalosporins in the range of 31.0%–57.0% and for ATM 44.0%–45.5%, indicating a high rate of ESBL-producing strains [17], which underlines the global health problem of cephalosporin resistance [18]. Previous SMART surveilence results found ESBL rates of 46.3%–49.1% for *E. coli* and 25.6%–26.8% for *K. pneumoniae * [13]. In the 2021 CHINET surveillance, resistance to third-generation cephalosporins was detected in 55.6% of *E. coli* and 43.8% of *K. pneumoniae*, also indicating the high ESBL prevalence in China [14]. The resistance rates for the fluoroquinolone LVX (36.6%–56.7%) were in a similar range to cephalosporins in this study, which might reflect the overuse of fluoroquinolones, especially since the development of cephalosporin-resistance [19, 20]. They were similar to the rates reported by the 2021 CHINET surveillance of LVX resistance detected as 53.6% for *E. coli* and 28.3% for* K. pneumoniae *isolates [14]. One approach to overcome cephalosporin resistance is the use of combination of a β-lactam and a β-lactamase inhibitor [21], such as tazobactam, which led to essentially reduced resistance rates of about 10% for otherwise cephalosporin-resistant *E. coli* and *K. pneumoniae* in the present study. Of concern is the rising resistance rate of *K. pneumoniae* to carbapenems*, *which reached25%–29% in the present study and was similar to the rate of about 25% found in the 2021 CHINET surveillance study [14].

Resistance rates for COL were generally low, but susceptibility breakpoints have been abolished in recent CLSI guidelines. Treatments with COL should be applied with maximum renally adjusted doses, since the previous MIC of 2 μg/mL could not be achieved in 50% of patients with normal renal functions and acute kidney injury occurs frequently with conventional doses. Recommendations include strongly preferred alternative drugs for active or combination treatments [22, 23]. Also, TOB is not commonly used in China, which might explain the low resistance rates found in the present study.

*P. aeruginosa* was the third most common pathogen detected and the most common Gram-negative pathogen found in RTI. It exhibited > 30% resistance to traditional antipesudomoas antibiotics, including CAZ, FEP, TZP, IPM, MEM and LVX. These results are similar to those reported in a recent SMART study that investigated *P. seudomonas* resistance to antibiotics in China [24].

This present survey included only isolates collected in EDs, since patients admitted to EDs are frequently candidates for urgent empiric antibiotic treatment, which should be administered according to the site of infection and the clinical severity of symptoms [25]. The characteristics of infected patients and physicians' routine treatments in the ED differed compared to other departments. This is because the infected site may initially not be clearly defined, patients cannot be observed for a long period of time, and etiological evidence is rarely available; doctors and nurses in EDs also have a very heavy workload. It is therefore convenient to use drugs which only need administration once a day to ED patients, including ETP once a day, AMK intramuscular injection, as well as LVX once a day, which can be sequenced simultaneously.

In order to offer guidelines for chosing an empiric treatment, organ specific therapy based on pathogen distribution differences and their antibiotic resistance variations have been developed [6, 8]. Of these, OSIWA was first applied for HA and CA infections of different intra-abdominal organs [9]. In the present study, we expanded the evaluation of OSIWA for the empirical treatment of IAI also to UTI, BSI and RTI. When considering the distribution of pathogens at a single infection site – for example *E. coli *caused more than 50% of all IAI and more than 60% of all UTI – whereas for RTI infections *E. coli *was the pathogen in < 15% of all cases (Fig. 3). The OSIWA shown in Fig 5 indicate the probabilities of successful empiric drug treatment for single infections sites. For RTI and IAI the choice of empirical antibiotics with expected efficacy was limited, but for UTI and BSI more antibiotics are available.

Limitations of the present survey were the relatively low sample numbers since only isolates from EDs have been included, which have had an influence on the resistance patterns reported and combination treatments with β-lactams, aminoglycoside and fluoroquinolone were not considered. In addition, since in the SMART data collection detailed data regarding resistance mechanisms are not included, susceptibility test results only can infer the mechanism of ESBL production in cephalosporin but not in carbapenem resistance.

## Conclusions

The pathogens isolated from BSI, IAI, UTI and RTI between 2016 and 2019 in Chinese EDs were mainly *E. coli, K. pneumoniae* and *P. aeruginosa*,but therewere considerable resistance pattern differences as well as organ distributions between the bacterial strains. OSIWA determinations led to organ specific antibiotic drug effectiveness patterns, which should help to guide the choice of suitable empirical antibiotic treatments, especially for urgent infected cases in EDs.

### Supplementary Information

**Supplementary Material 1.**

## Data Availability

The SMART database is not public and is only accessible to SMART investigators, but the data that support the findings of this study are directly available from MSD China or from the corresponding author Yunsong Yu upon reasonable request and with permission of MSD China.

## Abbreviations

AMK: amikacin
ATM: aztreonam
BSIs: blood stream infections
CA: community acquired
CAZ: ceftazidime
CHINET: China Antimicrobial Surveillance Network
COL: colistin
CRO: ceftriaxone
EDs: emergency departments
ETP: ertapenem
FEP: cefepime
FOX: cefoxitin
HA: hospital acquired
IAI: intra-abdominal infections
IPM: imipenem
LVX: levofloxacin
MEM: meropenem
MICs: minimum inhibitory concentrations
OSWIAs: organ-specific weighted incidence antibiograms
RTI: respiratory tract infections.
SMART: Study for Monitoring Antimicrobial Resistance Trends
TOB: tobramycin
TZP: piperacillin–tazobactam
UTI: urinary tract infections
