# Supplementary material for: Antibiotic susceptibility patterns and trends of the gram-negative bacteria isolated from the patients in the emergency departments in China: results of SMART 2016–2019
Source: BMC Infect Dis. 2024 May 17;24:501. doi: 10.1186/s12879-024-09294-0 (PMC11102128; doi:10.1186/s12879-024-09294-0)
Supplement: Supplementary file 1 — Supplementary Material 1. [file 12879_2024_9294_MOESM1_ESM.docx]

**Supplementary Table 1** Information on the 18 included hospitals

| Name of the hospital | Province | City | Number of isolates collected, n (%) | Number of common beds | Patients per year |
| --- | --- | --- | --- | --- | --- |
| The First Affiliated Hospital, Sun Yat-sen University | Guangdong | Guangzhou | 35 (5.3) | 3,888 | 4,800,000 |
| The First Affiliated Hospital, Zhejiang University School of Medicine | Zhejiang | Hangzhou | 2 (0.3) | 3,500 | 3,969,000 |
| The Second Affiliated Hospital of Nanchang University | Jiangxi | Nanchang | 16 (2.4) | 2,900 | 2,000,000 |
| Beijing Chao-Yang Hospital, Capital Medical University | Beijing | Beijing | 6 (0.9) | 1,900 | 3,800,000 |
| Peking Union Medical College Hospital | Beijing | Beijing | 82 (12.5) | > 2000 | 3,600,000 |
| Haikou People’s Hospital, Haikou Affiliated Hospital of Central South University Xiangya School of Medicine | Hainan | Haikou | 39 (5.9) | 1,950 | 1,090,000 |
| Jilin Province People’s Hospital | Jilin | Changchun | 149 (22.7) | 2,098 | 2,600,000 |
| Nanjing General Hospital of Nanjing Military Command | Jiangsu | Nanjing | 12 (1.8) | 2,000 | 2,310,000 |
| First Affiliated Hospital of Kunming Medical University, No.1 School of Clinical Medicine, Kunming Medical University | Yunnan | Kunming | 11 (1.7) | 4,000 | 2,950,000 |
| Shandong Provincial Hospital Affiliated to Shandong First Medical University | Shandong | Jinan | 60 (9.1) | > 3,500 | 4,420,000 |
| Huashan Hospital, Fudan University | Shanghai | Shanghai | 19 (2.9) | 1,216 | 4,000,000 |
| Zhongshan Hospital, Fudan University | Shanghai | Shanghai | 33 (5.0) | 2,005 | 4,000,000 |
| Sichuan Academy of Medical Sciences, Sichuan Provincial People's Hospital | Sichuan | Chengdu | 11 (1.7) | 4,132 | 4,310,000 |
| Sir Run Run Shaw Hospital, Zhejiang University School of Medicine | Zhejiang | Hangzhou | 74 (11.3) | 2,400 | 2,224,000 |
| The Affiliated Hospital of Qingdao University | Shandong | Qingdao | 90 (13.7) | 5,186 | 6,000,000 |
| The Hospital Group of the First Affiliated Hospital of CQMU, The First Affiliated Hospital Chongqing Medical University | Chongqing | Chongqing | 8 (1.2) | 3,200 | 3,407,700 |
| The Fourth Affiliated Hospital of Harbin Medical University | Heilongjiang | Harbin | 4 (0.6) | 3,200 | Unknown |
| Tongji Hospital, Tongii Medical College of HUST | Hubei | Wuhan | 5 (0.8) | > 5,000 | 6,440,000 |

**Supplementary Table 2** Composition of main bacterial flora in different infection sites in EDs

| Organism | BSI | IAI | UTI | RTI |
| --- | --- | --- | --- | --- |
| *E. coli* | 59 (48.4%) | 123 (58.6%) | 81 (72.3%) | 20 (9.6%) |
| *K. pneumoniae* | 30 (24.6%) | 45 (21.4%) | 13 (11.6%) | 82 (39.4%) |
| *P. aeruginosa* | 9 (7.4%) | 13 (6.2%) | 6 (5.4%) | 83 (39.9%) |
| *P. mirabilis* | 3 (2.5%) | 5 (2.4%) | 6 (5.4%) | 3 (1.4%) |
| *K. aerogenes* | 6 (4.9%) | 2 (1.0%) | 0 (0.0%) | 7 (3.4%) |
| *E. cloacae* | 2 (1.6%) | 5 (2.4%) | 2 (1.8%) | 2 (1.0%) |
| *S. marcescens* | 3 (2.5%) | 1 (0.5%) | 1 (0.9%) | 5 (2.4%) |
| *K. oxytoca* | 3 (2.5%) | 3 (1.4%) | 0 (0.0%) | 2 (1.0%) |
| Other | 7 (5.7%) | 13 (6.2%) | 3 (2.7%) | 4 (1.9%) |

Abbreviations: BSI, blood steam infection; ED, emergency department; IAI, intra-abdominal infection; RTI, respiratory tract infection; UTI, urinary tract infection.

**Supplementary Table 3** Composition ratio of Gram-negative bacteria from 2016 to 2019

|  | *E. coli*  n (%) | *K. pneumoniae*  n (%) | *P. aeruginosa*  n (%) | *P. mirabilis*  n (%) | *K. aerogenes*  n (%) | *E. cloacae*  n (%) | *S. marcescens*  n (%) | *K. oxytoca*  n (%) | Others  n (%) | All  N |
| --- | --- | --- | --- | --- | --- | --- | --- | --- | --- | --- |
| 2016 | 57 (45.2) | 31 (24.6) | 21 (16.7) | 5 (4.0) | 2 (1.6) | 0 (0) | 1 (0.8) | 2 (1.6) | 7 (5.6) | 126 |
| 2017 | 79 (47.3) | 42 (25.1) | 26 (15.6) | 4 (2.4) | 2 (1.2) | 2 (1.2) | 3 (1.8) | 2 (1.2) | 7 (4.2) | 167 |
| 2018 | 69 (36.1) | 54 (28.3) | 35 (18.3) | 3 (1.6) | 6 (3.1) | 8 (4.2) | 5 (2.6) | 3 (1.6) | 8 (4.2) | 191 |
| 2019 | 79 (45.9) | 44 (25.6) | 30 (17.4) | 5 (2.9) | 5 (2.9) | 1 (0.6) | 2 (1.2) | 1 (0.6) | 5 (2.9) | 172 |
| Total | 284 (43.3) | 171 (26.1) | 112 (17.1) | 17 (2.6) | 15 (2.3) | 11 (1.7) | 11 (1.7) | 8 (1.2) | 27 (4.1) | 656 |

**Supplementary Table 4** Comparison of the composition ratio of microbiota in different infected organs and age groups

|  | BSI | | | IAI | | | UTI | | | RTI | | |
| --- | --- | --- | --- | --- | --- | --- | --- | --- | --- | --- | --- | --- |
|  | < 39 years | 40-59 years | ≥ 60 years | < 39 years | 40-59 years | ≥ 60 years | < 39 years | 40-59 years | ≥ 60 years | < 39 years | 40-59 years | ≥ 60 years |
| *E. coli* | 3 (42.9%) | 10 (28.6%) | 46 (57.5%) | 35 (66.0%) | 35 (66.0%) | 53 (51.0%) | 17 (73.9%) | 21 (77.8%) | 43 (69.4%) | 2 (9.5%) | 4 (7.1%) | 14 (10.7%) |
| *K. pneumoniae* | 2 (21.2%) | 14 (40.0%) | 14 (17.5%) | 7 (13.2%) | 10 (18.9%) | 28 (26.9%) | 4 (17.4%) | 4 (14.8%) | 5 (8.1%) | 8 (38.1%) | 23 (41.1%) | 51 (38.9%) |
| *P. aeruginosa* | 1 (14.3%) | 3 (8.6%) | 5 (6.3%) | 4 (7.5%) | 2 (3.8%) | 7 (6.7%) | 1 (4.3%) | 0 (0.0%) | 5 (8.1%) | 7 (33.3%) | 24 (42.9%) | 52 (39.7%) |
| *P. mirabilis* | 0 (0.0%) | 1 (2.9%) | 2 (2.5%) | 2 (3.8%) | 1 (1.9%) | 2 (1.9%) | 1 (4.3%) | 0 (0.0%) | 5 (8.1%) | 1 (4.8%) | 1 (1.8%) | 1 (0.8%) |
| *K. aerogenes* | 0 (0.0%) | 4 (11.4%) | 2 (2.5%) | 0 (0.0%) | 0 (0.0%) | 2 (1.9%) | 0 (0.0%) | 0 (0.0%) | 0 (0.0%) | 1 (4.8%) | 1 (1.8%) | 5 (3.8%) |
| *E. cloacae* | 0 (0.0%) | 1 (2.9%) | 1 (1.3%) | 1 (1.9%) | 1 (1.9%) | 3 (2.9%) | 0 (0.0%) | 1 (3.7%) | 1 (1.6%) | 0 (0.0%) | 0 (0.0%) | 2 (1.5%) |
| *S. marcescens* | 0 (0.0%) | 0 (0.0%) | 3 (3.8%) | 1 (1.9%) | 0 (0.0%) | 0 (0.0%) | 0 (0.0%) | 0 (0.0%) | 1 (1.6%) | 1 (4.8%) | 1 (1.8%) | 3 (2.3%) |
| *K. oxytoca* | 0 (0.0%) | 1 (2.9%) | 2 (2.5%) | 0 (0.0%) | 1 (1.9%) | 2 (1.9%) | 0 (0.0%) | 0 (0.0%) | 0 (0.0%) | 1 (4.8%) | 1 (1.8%) | 0 (0.0%) |
| Other | 1 (14.3%) | 1 (2.9%) | 5 (6.3%) | 3 (5.7%) | 3 (5.7%) | 7 (6.7%) | 0 (0.0%) | 1 (3.7%) | 2 (3.2%) | 0 (0.0%) | 1 (1.8%) | 3 (2.3%) |
| Total | 7 (5.7%) | 35 (28.7%) | 80 (65.6%) | 53 (25.2%) | 53 (25.2%) | 104 (49.5%) | 23 (20.5%) | 27 (24.1%) | 62 (55.4%) | 21 (10.1%) | 56 (26.9%) | 131 (63.0%) |

Abbreviations: BSI, blood steam infections; IAI, intra-abdominal infection; RTI, respiratory tract infection; UTI, urinary tract infection.

**Supplementary Table 5** Isolation (detection rate) of carbapenem-resistant, quinolone-resistant, third-generation cephalosporin-resistant *E. coli*, *K. pneumoniae* and *P. aeruginosa* from 2016 to 2019

|  | 2016 | 2017 | 2018 | 2019 | Total |
| --- | --- | --- | --- | --- | --- |
| ***E. coli* (n)** | **57** | **79** | **69** | **79** | **284** |
| Carbapenem-resistant, n (%) | 5 (8.8) | 4 (5.1) | 2 (2.9) | 1 (1.3) | 12 |
| Quinolone-resistant, n (%) | 35 (61.4) | 43 (54.4) | 39 (56.5) | 44 (55.7) | 161 |
| Third-generation cephalosporin-resistant, n (%) | 36 (63.2) | 35 (44.3) | 36 (52.2) | 43 (54.4) | 150 |
| ***K. pneumoniae* (n)** | **31** | **42** | **54** | **44** | **171** |
| Carbapenem-resistant, n (%) | 11 (35.5) | 13 (31.0) | 10 (18.5) | 15 (34.1) | 49 |
| Quinolone-resistant, n (%) | 14 (45.2) | 20 (47.6) | 19 (35.2) | 16 (36.4) | 69 |
| Third-generation cephalosporin-resistant, n (%) | 3 (9.7) | 10 (23.8) | 13 (24.1) | 4 (9.1) | 30 |
| ***P. aeruginosa* (n)** | **21** | **26** | **35** | **30** | **112** |
| Carbapenem-resistant, n (%) | 11 (52.4) | 10 (38.5) | 16 (45.7) | 9 (30.0) | 46 |
| Quinolone-resistant, n (%) | 6 (28.6) | 12 (46.2) | 15 (42.9) | 8 (26.7) | 41 |
| Third-generation cephalosporin-resistant, n (%) | 3 (14.3) | 4 (15.4) | 1 (2.9) | 4 (13.3) | 12 |

**Supplementary Table 6** Differences in weighted drug susceptibilities of antibiotics at different organ and infection sites

|  | AMK | COL# | MEM | IPM | ETP | TOB | TZP | FOX | CAZ | LVX | FEP | ATM | CRO |
| --- | --- | --- | --- | --- | --- | --- | --- | --- | --- | --- | --- | --- | --- |
| **BSI** | 118 (96.7%) | 108 (88.5%) | 114 (93.4%) | 111 (91.0%) | 102 (90.3%) | 9 (100.0%) | 107 (87.7%) | 83 (73.5%) | 81 (66.4%) | 70 (57.4%) | 78 (63.9%) | 74 (60.7%) | 62 (54.9%) |
| **IAI** | 199 (94.8%) | 189 (90.0%) | 192 (91.4%) | 184 (87.6%) | 176 (89.3%) | 3 (75%) | 177 (84.3%) | 119 (60.4%) | 137 (65.2%) | 47 (22.4%) | 125 (59.5%) | 120 (57.1%) | 96 (48.7%) |
| IAI: Abscess | 26 (100.0%) | 24 (92.3%) | 25 (96.2%) | 24 (92.3%) | 24 (96.0%) | - | 22 (84.6%) | 14 (56.0%) | 18 (69.2%) | 6 (23.2%) | 15 (57.7%) | 14 (53.8%) | 12 (48.0%) |
| IAI: Appendix | 97 (97.0%) | 90 (90.0%) | 94 (94.0%) | 90 (90.0%) | 87 (92.6%) | 1 (100.0%) | 89 (89.0%) | 62 (66.0%) | 67 (67.0%) | 18 (18.0%) | 58 (58.0%) | 57 (57.0%) | 44 (46.8%) |
| IAI: Gall Bladder | 27 (93.1%) | 25 (86.2%) | 24 (82.8%) | 24 (82.8%) | 22 (84.6%) | - | 21 (72.4%) | 14 (53.8%) | 18 (62.1%) | 7 (24.1%) | 17 (58.6%) | 15 (51.7%) | 12 (46.2%) |
| IAI: Liver | 13 (86.7%) | 13 (86.7%) | 14 (93.3%) | 12 (80.0%) | 13 (86.7%) | - | 15 (100.0%) | 9 (60.0%) | 11 (73.3%) | 4 (26.3%) | 13 (86.7%) | 11 (73.3%) | 10 (66.7%) |
| IAI: Other | 0 (0.0%) | 1 (100.0%) | 0 (0.0%) | 0 (0.0%) | 0 (0.0%) | - | 0 (0.0%) | 0 (0.0%) | 0 (0.0%) | 0 (0.0%) | 0 (0.0%) | 0 (0.0%) | 0 (0.0%) |
| IAI: Pancreas | 2 (100.0%) | 2 (100.0%) | 2 (100.0%) | 2 (100.0%) | 1 (100.0%) | 1 (100.0%) | 1 (50.0%) | 1 (100.0%) | - | 2 (100.0%) | - | - | - |
| IAI: Peritoneal Fluid | 33 (91.7%) | 33 (91.7%) | 32 (88.9%) | 31 (86.1%) | 28 (82.4%) | 1 (100.0%) | 29 (80.6%) | 19 (55.9%) | 23 (63.9%) | 10 (27.9%) | 22 (61.1%) | 23 (63.9%) | 18 (52.9%) |
| IAI: Stomach | 1 (100.0%) | 1 (100.0%) | 1 (100.0%) | 1 (100.0%) | 1 (100.0%) | - | - | - | - | - | - | - | - |
| **UTI** | 104 (92.9%) | 98 (87.5%) | 104 (92.9%) | 95 (84.8%) | 95 (89.6%) | 2 (100%) | 92 (82.1%) | 75 (70.8%) | 72 (64.3%) | 27 (24.1%) | 62 (55.4%) | 63 (56.3%) | 53 (50%) |
| UTI: Ureter | 3 (100.0%) | 2 (66.7%) | 3 (100.0%) | 2 (66.7%) | 3 (100.0%) | - | 3 (100.0%) | 3 (100.0%) | 3 (100.0%) | 1 (33.0%) | 2 (66.7%) | 2 (66.7%) | 2 (66.7%) |
| UTI: Urine | 101 (92.7%) | 96 (88.1%) | 101 (92.7%) | 93 (85.3%) | 92 (89.3%) | 2 (100.0%) | 89 (81.7%) | 72 (69.9%) | 69 (63.3%) | 26 (23.3%) | 60 (55.0%) | 61 (56.0%) | 51 (49.5%) |
| **RTI** | 173 (83.2%) | 179 (86.1%) | 132 (63.5%) | 126 (60.6%) | 86 (68.8%) | 42 (85.7%) | 111 (53.4%) | 67 (53.6%) | 118 (56.7%) | 64 (30.8%) | 104 (50%) | 86 (41.4%) | 50 (40%) |
| RTI: Bronchoalveolar lavage | 7 (87.5%) | 7 (87.5%) | 3 (37.5%) | 4 (50.0%) | 2 (40.0%) | 3 (100.0%) | 3 (37.5%) | 1 (20.0%) | 5 (62.5%) | 4 (50.5%) | 5 (62.5%) | 2 (25.0%) | 1 (20.0%) |
| RTI: Endotracheal aspirate | 2 (100.0%) | 2 (100.0%) | 2 (100.0%) | 2 (100.0%) | - | - | 1 (50.0%) | - | 1 (50.0%) | 0 (0.0%) | 1 (50.0%) | - | - |
| RTI: Sputum | 162 (82.7%) | 169 (86.2%) | 125 (63.8%) | 118 (60.2%) | 82 (69.5%) | 39 (84.8%) | 105 (53.6%) | 64 (54.2%) | 110 (56.1%) | 60 (30.1%) | 96 (49.0%) | 82 (41.8%) | 47 (39.8%) |
| RTI: Thoracentesis | 2 (100.0%) | 1 (50.0%) | 2 (100.0%) | 2 (100.0%) | 2 (100.0%) | - | 2 (100.0%) | 2 (100.0%) | 2 (100.0%) | 0 (0.0%) | 2 (100.0%) | 2 (100.0%) | 2 (100.0%) |
| **Unknown** | 4 (100.0%) | 3 (75.0%) | 3 (75.0%) | 3 (75.0%) | 2 (66.7%) | 1 (100.0%) | 3 (75.0%) | 0 (0.0%) | 3 (75.0%) | 1 (25.0%) | 3 (75.0%) | 3 (75.0%) | 1 (33.3%) |

Note: #, intermediate rate was shown for COL; -, no detection.

Abbreviations: AMK, amikacin; ATM, aztreonam; BSI, blood steam infections; CAZ, ceftazidime; CRO, ceftriaxone; ETP, ertapenem; FEP, cefepime; FOX, cefoxitin; IAI, intra-abdominal infections; IPM, imipenem; LVX, levofloxacin; MEM, meropenem; RTI, respiratory tract infections; TOB, tobramycin; TZP, piperacillin-tazobactam; UTI, urinary tract infections.
